# The combination of the entomopathogenic fungus *Metarhizium anisopliae *with the insecticide Imidacloprid increases virulence against the dengue vector *Aedes aegypti *(Diptera: Culicidae)

**DOI:** 10.1186/1756-3305-4-8

**Published:** 2011-01-25

**Authors:** Adriano R Paula, Aline T Carolino, Cátia O Paula, Richard I Samuels

**Affiliations:** 1 Department of Entomology and Plant Pathology, Universidade Estadual do Norte Fluminense Darcy Ribeiro, Campos dos Goytacazes RJ CEP 28013-602 Brazil

## Abstract

**Background:**

Dengue fever transmitted by the mosquito *Aedes aegypti*, is one of the most rapidly spreading insect borne diseases, stimulating the search for alternatives to current control measures. The dengue vector *A. aegypti *has received less attention than anophelene species, although more than 2.5 billion people are at risk of infection worldwide. Entomopathogenic fungi are emerging as potential candidates for the control of mosquitoes. Here we continue our studies on the pathogenicity of the entomopathogenic fungus *Metarhizium anisopliae *against adult *A. aegypti *females. With the aim of further reducing mean survival times of *A. aegypti *exposed to fungus impregnated surfaces, a sub-lethal concentration of the neonicotinoid insecticide Imidacloprid (IMI) was added to fungal suspensions.

**Results:**

A sub-lethal concentration of IMI that did not significantly alter the daily survival rates or mean survival percentages of mosquitoes was identified to be 0.1 ppm. This sub-lethal concentration was combined with *M. anisopliae *conidia (1 × 10^9 ^conidia mL^-1^). Both the combined treatment and the conidia alone were able to reduce the survival of *A. aegypti *compared with untreated or IMI treated mosquitoes. Importantly, mosquito survival following exposure to the combined treatment for 6 and 12 hrs was significantly reduced when compared with mosquitoes exposed to conidia alone.

**Conclusions:**

This is the first time that a combination of an insecticide and an entomopathogenic fungus has been tested against *A. aegypti*. Firstly, the study showed the potential of IMI as an alternative to the currently employed pyrethroid adulticides. Secondly, as an alternative to applications of high concentrations of chemical insecticides, we suggest that adult *A. aegypti *could be controlled by surface application of entomopathogenic fungi and that the efficiency of these fungi could be increased by combining the fungi with ultra-low concentrations of insecticides, resulting in higher mortality following relatively short exposure times.

## Background

During the 2008 Dengue epidemic in Brazil, 743,517 cases were registered, falling to 387,158 cases in 2009, resulting in 98 deaths from dengue haemorrhagic fever and 58 deaths for Dengue related complications [1]. Up until October 2010, 936,260 cases had been notified with 592 Dengue related deaths [2]. With the development of a multi-sorotype vaccine still a distant goal, the only means of reducing infection rates is to control the insect vector.

Conventional mosquito control measures in Brazil consist of year-round vigilance, continuous applications of chemical or biological larvicides, elimination of breeding sites and during Dengue outbreaks, spray application of insecticides for reduction of adult mosquito populations [3]. The efficiency of year-round interventions has been recently questioned by Luz *et al. *[4], who demonstrated by mathematical modeling that interventions with chemical insecticides for larval and adult *A. aegypti *control need only be performed at the start of the Dengue season for optimal results. Furthermore, adult control using insecticide treated surfaces and lethal ovitraps were more effective than conventional adult control measures (ultra-low volume insecticide spraying). However, a faster resistance evolution was predicted for these new approaches [4].

Studies have shown the widespread resistance of *A. aegypti *larvae to the organophosphate temephos throughout Brazil [5]. Resistance of adult *A. aegypti *to the currently used pyrethroids such as cypermethrin and deltamethrin has also been reported [6]. Therefore new approaches are urgently needed.

Chemical insecticides used in vector control interventions have undoubtedly saved millions of human lives and will continue to be of utmost importance for reducing incidences of insect transmitted diseases. However, evolution of resistance is a major concern, reducing the effectiveness of currently available insecticides. Novel lines of research are being undertaken, which include diverse approaches such as the possible release of genetically modified insects refractory to infection by the microorganisms responsible for causing diseases [7,8], genetically modifying insects to increase susceptibility to microbial control agents [9] and the development of new insecticidal compounds (natural and synthetic) in the continuous fight against insecticide resistant vector populations. The potential of fungal pathogens for the control of the adult stage of the malaria mosquito *Anopheles *was first highlighted by two high impact research papers [10,11]. Over the last year further studies of the possible use of entomopathogenic fungi for the control of malaria mosquitoes have been documented [e.g. [12-15]]. The dengue vector *Aedes aegypti *has not received the same level of attention, although it is also a possible target for entomopathogenic fungal control agents.

Entomopathogenic fungi such as *Metarhizium *and *Beauveria *are currently used for the control of agricultural and forest pests on a world wide basis [16]. Their "rediscovery" for use against vectors has been stimulated by the fact that it is not necessary to kill a vector insect to reduce vectorial capacity. As reiterated by Thomas & Read [13], successful malarial parasite transmission is correlated with mosquito longevity: the vector is required to survive for at least the 2 week incubation period of the parasite. Therefore even small alterations in longevity can result in dramatic effects on transmission. Moreover, "non-lethal" effects of microbial infections could also reduce vectorial capacity by reducing propensity to feed, fecundity, flight/dispersal capacity and predator escape responses [10,17].

We are currently investigating the possibility of using fungus impregnated black cotton cloths for the control of adult *A. aegypti *[18] by placing these cloths in strategic positions where *A. aegypti *females are known to rest in human dwellings. Black cloths attract mosquitoes during resting periods as they can remain undetected on these surfaces. Impregnating these cloths with entomopathogenic fungi would act as lethal baits.

This type of technique has been tested once under field conditions in Africa for the control of the malaria vector *Anopheles gambiae *[11]. Fungus impregnated black cotton sheets hung from the ceilings of dwellings caused a 23% infection rate. This infection rate, when considered in a model for malarial transmission, predicted a 75% reduction in malarial transmission. This prediction does not take into account the other "positive" effects due to the fungi infecting the mosquito, such as the reduction in blood feeding and inhibition of malarial parasite development [10]. Reduced vectorial capacity of *A. aegypti *could also be expected following infection by entomopathogenic fungi, however the incubation period for the Dengue arbovirus in the mosquito is between 7 to 10 days [19], a shorter period than that of *Plasmodium *development in *Anopheles *(14 days), indicating that highly virulent isolates of entomopathogenic fungi may need to be deployed against Dengue mosquitoes.

The mosquito *A. aegypti *is an interesting candidate for a fungal control program as it has evolved an intimate relationship with man, and can be described as an "urbanized" vector. Dengue could be classified as a man-made problem, with increasing incidences of epidemics directly correlated to high inner city human population densities, the uncontrollable increase in the use of non-biodegradable plastic packaging, deficient local government trash collection and lack of adequate sewage systems. On one hand the close proximity with urban conurbations means that *A. aegypti *is a highly dangerous vector whilst on the other hand limiting the areas that need to be targeted during interventions in order to control this insect.

Knowledge of insect behavior is also paramount in the planning of control strategies. For example, the emerging *A. aegypti *females seek blood meals during the early morning or early evening. Thus, the use of insecticide impregnated bed-nets, highly successful for the control of malaria vectors [20], appears not to be an option for *A. aegypti *control. Interestingly, *A. aegypti *populations were significantly reduced in field trials where window curtains had been treated with insecticide [21]. Similar application technologies for entomopathogenic fungi could offer an alternative to conventional control measures. However, window curtains impregnated with fungi would have to take into consideration the fact that these microorganisms are highly susceptibility to the damaging effects of UV [22], although strategically placing fungus-impregnated cloths within dwellings out of direct sunlight could be tested for *A. aegypti *control.

The combination of low concentrations of the insecticide Imidacloprid with entomopathogenic fungi has been the subject of previous studies by our group aimed at improving efficiency of the fungi for the control of agricultural pest species [23,24] and it has been shown that Imidacloprid has no effect on conidial germination [23]. IMI is a systemic insecticide of the neonicotinoid group, with contact and oral toxicity. The site of action of this insecticide is the nicotinic acetylcholine receptor, therefore acting as a neurotoxin [25]. It was also found to act synergistically with *Beauveria bassiana *and *Metarhizium anisopliae *for the control of the Coleopteran *Diaprepes abbreviatus *[26].

With the aim of increasing the efficacy of an entomopathogenic fungus for the control of *A. aegypti *adult females, experiments were carried out to first determine the sub-lethal concentration of Imidacloprid to be combined with a highly virulent isolate of *M. anisopliae *(LPP 133). Experiments were then performed to investigate the minimum time necessary to significantly lower daily survival rates of *A. aegypti *exposed to the combined agents with the future aim of reducing *A. aegypti *adult populations and thus reducing the incidence of Dengue.

## Results

**Figure 1:**
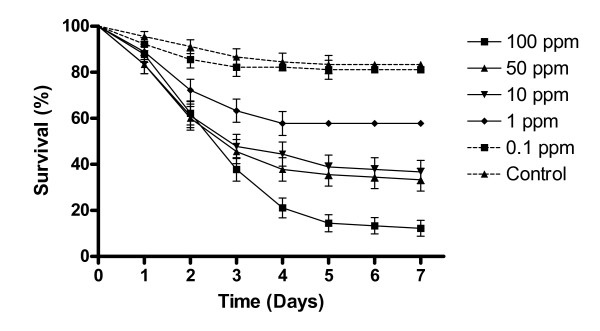
**Survival curves of female *Aedes aegypti *exposed to five concentrations of Imidacloprid**. Note: The control survival curve is the mean of all control groups (5) used for each IMI concentration. Results are the means of three experiments for each concentration of IMI tested with 30 insects used per experiment. Data points without standard error bars had errors equal to that of the previous data point.

The sub-lethal concentration of IMI to be used in subsequent experiments was determined using the concentration-response curve of female *A. aegypti*. The LC_50 _value was 4.04 ppm (confidence limits 0.88-15.3). The concentration of IMI used was negatively correlated with survival of *A. aegypti *(Figure 1). The only concentration tested that did not result in significantly different survival to the untreated controls (84.6% ± 0.95) was 0.1ppm with 82.3% (± 1.97) of mosquitoes remaining alive at the end of the experiment.

**Figure 2:**
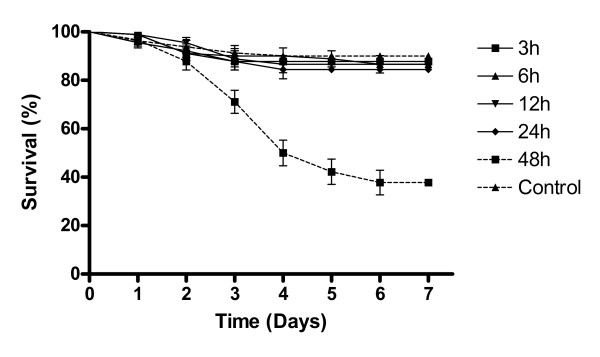
**Survival curves of female *Aedes aegypti *exposed to 0.1 ppm Imidacloprid for different time periods**. Note: The control survival curve is the mean of all control groups (5) used for each time period. Results are the means of three experiments for each time period tested, with 30 insects used per experiment. Data points without standard error bars had errors equal to that of the previous data point.

In order to verify the possible effect of 0.1 ppm IMI on mosquito survival following different time periods, insects were exposed to IMI for 3 to 48 h (Figure 2). All exposure times were not significantly different from the control values except for a 48 h exposure [F_(5,17) _= 87.69 *p *< 0.01], which reduced mean survival to 37.7% (± 2.69). Survival curve comparisons using the Log-Rank test also confirmed this result (*p *< 0.0001). Therefore exposure times longer than 24 h were avoided in all future experiments.

**Figure 3:**
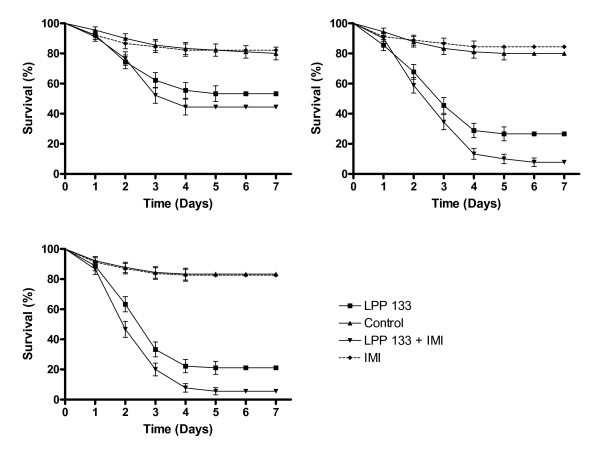
**Survival curves of female *Aedes aegypti *exposed to 0.1 ppm Imidacloprid, LPP 133 alone and Imidacloprid + LPP 133 during three different time periods: (A) 3 h; (B) 6 h; (C) 12 h**. Note: Results are the means of three experiments for each treatment tested, with 30 insects used per experiment. Data points without standard error bars had errors equal to that of the previous data point.

When female mosquitoes were exposed to a combination of 0.1 ppm IMI plus the entomopathogenic fungus *M. anisopliae*, a reduction in survival rates were observed when compared to the use of the fungus alone. The survival curves for experiments where the mosquitoes were exposed to IMI, fungus alone and combinations of IMI + fungus for three different time periods (3, 6 and 12 h) are shown in Figure 3. Treatments with fungus alone and IMI + fungus for 3 h resulted in moderate reductions in survival rates (51% and 44% respectively), significantly different from control survival (83% survival; F_(3,11) _= 5.11 *p *< 0.05), however, there was no significant difference between fungus alone and IMI + fungus (Table 1; *p *> 0.05). The daily survival rates for insects exposed to the different treatments for 6 h are shown in Figure 3B. Lower percentage survival was observed here and importantly a significant difference between fungus and IMI + fungus, with 26% of LPP 133 treated females surviving and only 11% survival of LPP 133 + IMI treated females (Table 1; F_(3,11)_= 172.05, *p *< 0.01), although there was no significant difference between survival curves when using the Log-Rank test. A twelve hour exposure of insects to the different treatments showed a further decline in survival rates and the mean percentage survival was significantly different when comparing fungus and IMI + fungus (Table 1; F_(3,11) _= 105.17, *p *< 0.01). Interestingly, following a 12 h exposure, there was a significant difference between the survival curves. The S_50 _value for IMI + fungus was estimated to be 2 days whilst exposure to the fungus alone resulted in a S_50 _value of 3 days (Table 1).

**Table 1:** Survival of mosquitoes exposed to Imidacloprid, fungus and Imidacloprid+fungus during three different time periods

Exposure time (h)	Treatments	% Survival ± SD	**S**_**50**_
			

	TWEEN	83.3 ± 2.85 a	ND

	IMI	85.5 ± 2.67 a	ND

3	FUNGUS	51.1 ± 6.01 b	ND

	IMI + FUNGUS	44.4 ± 8.25 b	ND

			

			

			

	TWEEN	80 ± 2.43 a	ND

	IMI	84.4 ± 2.82 a	ND

6	FUNGUS	26.6 ± 8.48 b	3

	IMI + FUNGUS	11.1 ± 11.34 c	3

			

			

	TWEEN	83.3 ± 2.67 a	ND

	IMI	85.5 ± 2.47 a	ND

12	FUNGUS	23.3 ± 10.6 b	3

	IMI + FUNGUS	7.77 ± 12.32 c	2

			

Results followed by the same letter indicate no significant differences when using Duncan's post-hoc test (5% probability). Values for S_50 _(mean survival time) were calculated using Kaplan-Meier survival analysis. ND = Not determined.

## Discussion

Fungi are potential candidates for the control of adult Dengue mosquitoes as confirmed in the current study and previously [18,27]. Sub-lethal concentrations of IMI when combined with LPP 133 significantly reduced insect survival rates following a 6 h exposure of adult female mosquitoes to the two agents and significantly reduced mean survival times from 3 to 2 days following a 12 h exposure. At first hand it would seem unlikely that mosquitoes would rest for 6 to 12 h on fungus impregnated surfaces in order to reap the benefit of the combined agents, however, it should be remembered that our aim is not necessarily to cause rapid mortality of the mosquito but reduce survival rates, even if by small amounts.

In experiments where mosquitoes are forced to rest on fungus impregnated surfaces, increasing exposure times from 15 min to 30 min resulted in a more rapid killing effect, with 30 min exposure times causing 100% mortality in nine days and a 15 min exposure time causing 100% mortality in fourteen days [14].

Studies of fungal infections of malaria mosquitoes have shown that relatively small changes in survival rates were sufficient to significantly reduce vectorial capacity [13]. Thus significant reductions in survival rates using LPP 133 + IMI could reflect in a decline of DENV transmission rates. This will remain to be proven in field trials. Another factor to be taken into consideration is the conidial concentration applied to surfaces onto which the mosquitoes rest and subsequently become infected. In our previous studies we selected a conidial concentration that caused 80-90% mortality over a seven day period [18], considering the incubation time of the Dengue virus. The concentration of conidia used is normally correlated to mortality rates, with higher mortality correlated to high conidial concentrations. However, ever increasing concentrations do not result in ever increasing mortality, with maximal reduction in survival rates seen when using 2 × 10^10 ^conidia m^-2 ^[14]. The economic viability of applying very high conidia concentrations should also be taken into account.

The strategy of combining insecticides with biological control agents is an interesting approach for controlling certain agricultural pest species, allowing the use of low concentrations of chemical insecticides, reducing the environmental impact of these chemicals and lowering the time taken for the pathogen to kill its host, a limiting factor in the acceptance of these agents. Insecticides have the capacity to increase stress and alter insect behavior that may lead to improved performance of entomopathogens [28,29].

The effects of the combination of insecticides and entomopathogenic fungi have been recently studied for the control of malaria mosquitoes. Mosquitoes pre-infected with *B. bassiana *or *M. anisopliae *showed a significant increase in mortality after exposure to permethrin when compared with mosquitoes exposed only to the insecticide, although mortality following exposure to the fungus alone was not evaluated in this experiment [15].

Another study investigated the effects of fungus impregnated window screens in combination with insecticide treated bed nets against *Culex quinquefasciatus *[30]. The only significant behavioral modification found was a reduction in blood feeding by *Cx. quinquefasciatus*, caused by the permethrin and *B. bassiana *treatments, although no additive effect was observed. *B. bassiana *did not repel blood foraging mosquitoes either in the laboratory or in the field [30].

IMI has never been tested before in combination with fungi for control of mosquitoes. IMI is not currently registered for use in mosquito control but has been tested previous against three mosquito species, including *A. aegypti *[31]. Neonicotinoids such as IMI were shown to be toxic to larval and adult *A. aegypti*, although IMI was only considered to be an interesting candidate for larval control [32]. From the results of the current study we consider that adult *A. aegypti *are highly susceptible to IMI, with an indirect unforced contact bioassay giving a LC_50 _value of 4 ppm.

Recently it has been shown that insecticide resistant populations of the malaria mosquito are equally susceptible to fungal infection as their baseline counterparts, and that fungal infection reduced the expression of resistance to the key public health insecticides permethrin and dichlorodiphenyltrichloroethane [15]. It has also been stated that pyrethroid resistant *Anopheles gambiae *were more susceptible to fungal infection that non-resistant strains [12], however as two different strains were used in these experiments is difficult to make a direct comparison.

Although we consider *M. anisopliae *LPP 133 to be highly virulent to female *A. aegypti*, it is important to take into consideration the necessity of further reducing vector survival, as the unknown factor in this putative control strategy is the length of time the mosquitoes will eventually rest on the fungus impregnated surfaces. With the aim of increasing the time that mosquitoes may rest on the fungus impregnated cloths, we attached an attractant (BG Lure™; Biogents Ltd.) to the cloths, however no increased mortality was observed (unpublished data).

The virulence of a putative biological control agent such as an entomopathogenic fungus selected for *A. aegypti *control is important as any small alteration in kill time could have consequences for the transmission of the Dengue virus. Interestingly, DENV infected *A. aegypti *became immuno-compromised due to down-regulated expression levels of numerous immune signaling molecules and antimicrobial peptides [33], leading us to speculate that these mosquitoes could be more susceptible to fungal attack than non-DENV infected insects, a further bonus to a fungal control strategy.

In this study, we investigated the possibility of combining an insecticide with an entomopathogenic fungus with the aim of reducing vectorial capacity by joint action of the two agents. It is known that no "silver bullet" solution is available for *A. aegypti *control and an Integrated Vector Management approach should therefore be adopted.

## Methods

### Insects

*A. aegypti *(Rockefeller strain) colonies were reared in cages at 25°C; 75% RH;16:8 L/D photoperiod and provided with a 10% sucrose solution. Insects were provided with blood meals by placing a mouse, immobilized in a wire mesh bag, in the adult mosquito cages (method approved by the University of North Fluminense Ethical Committee). Following the blood meal, oviposition occurred in beakers half filled with water and lined with filter paper placed in adult cages. Egg eclosion was stimulated by total immersion of the filter paper in water to which mouse food had been previously added (24 h) to reduce oxygen levels.

Larvae were maintained in plastic trays and fed on minced commercial mouse food until reaching the pupal stage. Pupae were separated into water filled beakers and transferred to cages before adult emergence. Recently hatched (2-3 day old) females were used for all experiments.

### Concentration response curve for mosquitoes exposed to Imidacloprid

The commercial insecticide Confidor^® ^(Bayer, Brazil) which is composed of 70% Imidacloprid (IMI) and 30% inert materials was used here. Adult female *A. aegypti *were exposed to filter papers impregnated with 0.1, 1, 10, 50 and 100 ppm IMI dissolved in sterile distilled water + 0.05% Tween 80. One mL of IMI was sprayed onto each side of a piece of filter paper (8 × 6 cm) using a Potter tower (Burkart Ltd. UK). Following spraying the filter papers were left to dry for 16 h before being placed in plastic pots covered with netting (12 cm diameter × 7 cm high), to which mosquitoes were then placed. The filter paper was positioned upright in the pot. Thus, the total time the mosquitoes spent resting on the filter paper can not be determined by this method as mosquitoes could choose not to land on the filter paper (see Additional File 1; Figure S1). Adult females were exposed to the filter papers for 24 h, following which time the mosquitoes were transferred to clean pots. The pots were maintained in an incubator at 25°C; 70 ± 10% RH; 12L:12D. Mosquitoes were fed on 10% sucrose offered on filter paper discs placed on the netting surface. Survival of insects was determined on a daily basis for a 7 day period and dead insects were removed during observations.

It should be noted that these toxicity tests were not carried out using the WHO recommended protocol [34] as we adopted a method that allowed mosquitoes a choice of resting surfaces, considering that in the field, mosquitoes would not be forced to rest on insecticide or fungus impregnated surfaces.

### Effects of sub-lethal concentrations of IMI during different exposure times

A sub-lethal concentration of IMI, as determined in the previous experiment, was used to investigate the relationship between increasing exposure times of insects to insecticide impregnated filter papers on daily survival rates. In this experiment, mosquitoes were exposed to IMI impregnated filter papers for 2, 8, 16, 24 and 48 hours and survival monitored as stated above.

### Fungal Isolates and preparation of suspensions

An isolate of *M. anisopliae *from the collection of the Laboratory of Entomology and Plant Pathology at the State University of North Fluminense denominated LPP133 (originally isolated from a soil sample), was used in all experiments here as it had previously been demonstrated to have high virulence against adult *A. aegypti *[18]. Fungi were cultured on Sabouraud Dextrose Agar (Dextrose 10g; Peptone 2.5g; Yeast Extract 2.5g; Agar 20g in 1 L H_2_0) at 27°C for 15 days before being used in experiments. Fungal suspensions were initially prepared in Tween 80 (0.05% in sterile distilled water) and conidial concentration determined using a Neubauer hemocytometer. A standard concentration of 1 × 10^9 ^conidia mL^-1 ^was used in all experiments. Fungal suspensions were vortex mixed vigorously before applying 1 mL to each side of a piece of filter paper (8 × 6 cm) using a Potter spray tower giving a conidial coverage of 1.5 × 10^8 ^conidia cm^2 ^on each side of the filter paper. As was the case for exposure of mosquitoes to IMI, the actual time the insects spent resting on the fungus impregnated filter paper could not be determined.

### Exposure of insects to conidial suspensions formulated with IMI

*M. anisopliae *conidial suspensions were prepared with and without a sub-lethal concentration of 0.1 ppm IMI (see results section) and sprayed onto filter papers as stated above. Female mosquitoes were exposed to filter papers for 3, 6 or 12 hours, before being transferred to clean pots. Insect survival rates were monitored as stated above. Control groups were exposed to 0.1 ppm IMI and 0.05% Tween 80 treated filter papers.

All experiments were carried out three times with a minimum of 30 insects per treatment or control group. The homogeneity of the replicate experiments was determined using the Log Rank Test [35] at the 95% significance level and subsequently the results were pooled for survival curve analysis, mean percentage survival and standard deviation.

### Statistical Analysis

Probit analysis was used to calculate the LC_50 _of IMI for female *A. aegypti *and the sub-lethal concentration of IMI was verified by one-way analysis of variance and Duncan's *post-hoc *test when comparing control and insecticide exposed insect mortality. Mean percentage survival over a seven day period following treatments was calculated and the results compared using one-way analysis of variance and Duncan's *post-hoc *test. Survival curve comparisons were carried out using the Log-Rank test. Median survival time (*S*_50_) was calculated using Kaplan-Meier survival analysis.

## Competing interests

The authors declare that they have no competing interests.

## Authors' contributions

ARP carried out the experiments, participated in the design of the study and performed the statistical analysis. ATC and COP helped carry out experiments and maintained the insect colonies. RIS conceived the study, participated in its design, supervised the experiments and wrote the manuscript. All authors read and approved the final manuscript.

## Supplementary Material

Additional file 1 **Figure S1 Insecticide and fungal exposure system **Photograph showing the type of plastic pot used in insecticide testing and fungal infection. The filter paper shown here was impregnated by submersion in a conidial suspension for display purposes only. Mosquitoes released into the pot (plastic lid removed for clarity) had free access to resting sites not treated with fungi or insecticide. Click here for file

## References

[B1] Ministério da Saúde Secretaria de Vigilância Em Saúde Dengue No Brasil Informe Epidemiológico17/2009 http://www.dengue.org.br/boletimEpidemiologico_n026.pdf

[B2] Ministério da Saúde Programa Nacional de Controle da Dengue http://portal.saude.gov.br/portal/saude/visualizar_texto.cfm?idtxt=23614 accessed: 26/11/10

[B3] Donalísio MR Glasser CM Vigilância entomológica e controle de vetores do Dengue Rev Bras Epidemiol 2002 5 259 272

[B4] Luz PM Codeço CT Medlock J Struchiner CJ Valle D Galvani AP Impact of insecticide interventions on the abundance and resistance profile of *Aedes aegypti* Epidemiol Infect 2009 137 1203 1215 10.1017/S0950268808001799 19134235

[B5] Montella IR Martins AJ Viana-Medeiros PF Lima JB Braga IA Valle D Insecticide resistance mechanisms of Brazilian *Aedes aegypti *populations from 2001 to 2004 Am J Trop Med Hyg 2007 77 467 477 17827362

[B6] da-Cunha MP Lima JB Brogdon WG Moya GE Valle D Monitoring of resistance to the pyrethroid cypermethrin in Brazilian *Aedes aegypti *(Diptera: Culicidae) populations collected between 2001 and 2003 Mem Inst Oswaldo Cruz 2005 100 441 444 10.1590/S0074-02762005000400017 16113895

[B7] James AA Gene drive systems in mosquitoes: rules of the road Trends Parasitol 2005 21 64 67 10.1016/j.pt.2004.11.004 15664528

[B8] Marrelli MT Li C Rasgon JL Jacobs-Lorena M Transgenic malaria-resistant mosquitoes have a fitness advantage when feeding on Plasmodium-infected blood PNAS 2007 104 5580 5583 10.1073/pnas.0609809104 17372227 PMC1838510

[B9] Shin SW Kokoza V Lobkov I Raikhel AS Relish-mediated immune deficiency in the transgenic mosquito *Aedes aegypti* PNAS 2003 100 2616 2621 10.1073/pnas.0537347100 12594340 PMC151389

[B10] Blanford S Chan BHK Jenkins N Sim D Turner RJ Read AF Thomas MB Fungal pathogen reduces potential for malaria transmission Science 2005 308 1638 1641 10.1126/science.1108423 15947189

[B11] Scholte EJ Ng`habi K Kihonda J Takken W Paaijmans K Abdula S Killeen GF Knols BGJ An entomopathogenic fungus for control of adult African malaria mosquitoes Science 2005 308 1641 1642 10.1126/science.1108639 15947190

[B12] Howard AFV Koenraadt CJM Farenhorst M Knol BGJ Takken W Pyrethroid resistance in *Anopheles gambiae *leads to increased susceptibility to the entomopathogenic fungi *Metarhizium anisopliae *and *Beauveria bassiana* Malar J 2010 9 168 10.1186/1475-2875-9-168 20553597 PMC2898789

[B13] Thomas MB Read AF Can fungal biopesticides control malaria? Nature Rev: Microbiol 2007 5 377 383 10.1038/nrmicro1638 17426726

[B14] Mnyone LL Kirby MJ Lwetoijera DW Mpingwa1 MW Knols BGJ Takken W Russell TL Infection of the malaria mosquito, *Anopheles gambiae*, with two species of entomopathogenic fungi: effects of concentration, co-formulation, exposure time and persistence Malar J 2009 8 309 10.1186/1475-2875-8-309 20030834 PMC2808315

[B15] Farenhorst M Mouatchob JC Kikankieb CK Brookeb BD Hunt RH Thomas MB Koekemoerb LL Knols BGJ Coetzee M Fungal infection counters insecticide resistance in African malaria mosquitoes PNAS 2009 106 17443 17447 10.1073/pnas.0908530106 19805146 PMC2762667

[B16] Butt TM Jackson C Magan N Fungi as Biocontrol Agents: Progress, Problems and Potential CABI Int 2001 Wallingford, UK

[B17] Scholte EJJ Knols BGJ Takken W Infection of the malaria mosquito *Anopheles gambiae *with the entomopathogenic fungus *Metarhizium anisopliae *reduced blood feeding and fecundity J Invertebr Pathol 2006 91 43 49 10.1016/j.jip.2005.10.006 16376375

[B18] Paula AR Brito ES Pereira CR Carrera MP Samuels RI Susceptibility of adult *Aedes aegypti *(Diptera: Culicidae) to infection by *Metarhizium anisopliae *and *Beauveria bassiana*: prospects for Dengue vector control Biocont Sci Tech 2008 18 1017 1025 10.1080/09583150802509199

[B19] Salazar MI Richardson JH Sanchez-Vargas I Olson KE Beaty BJ Dengue virus type 2: replication and tropisms in orally infected *Aedes aegypti *mosquitoes BMC Microbiol 2007 7 9 10.1186/1471-2180-7-9 17263893 PMC1797809

[B20] Itoh T Evaluation of long-lasting insecticidal nets after 2 years household use Trop Med Inter Health 2005 10 1321 1326 10.1111/j.1365-3156.2005.01523.x 16359414

[B21] Kroeger A Lenhart AE Ochoa M Villegas E Levy M Alexander N McCall PJ Effective dengue vector control with curtains and water container covers treated with insecticide in Mexico and Venezuela: cluster randomised trials Brit Med J 2006 332 1247 1250 10.1136/bmj.332.7552.1247 16735334 PMC1471956

[B22] Morley-Davis J Moore D Prior C Screening of *Metarhizium *and *Beauveria *spp. conidia with exposure to simulated sunlight and a range of temperatures Mycol Res 1995 100 31 38 10.1016/S0953-7562(96)80097-9

[B23] Santos A Oliveira BL Samuels RI Selection of entomopathogenic fungi for use in combination with sub-lethal doses of imidacloprid: perspectives for the control of the leaf-cutting ant *Atta sexdens rubropilosa *Forel (Hymenoptera: Formicidae) Mycopathol 2007 163 233 240 10.1007/s11046-007-9009-8 17404893

[B24] Brito ES Paula AR Vieira LP Dolinski C Samuels RI Combining vegetable oil and sub-lethal concentrations of Imidacloprid with *Beauveria bassiana *and *Metarhizium anisopliae *against adult guava weevil *Conotrachelus psidii *(Coleoptera: Curculionidae) Biocont Sci Tech 2008 18 665 673 10.1080/09583150802195965

[B25] Elbert A Becker B Hartwig J Erdelen C Imidacloprid - a new systemic insecticide Pflanzenschutz-Nachrichten Bayer 1991 44 113 136

[B26] Quintela ED McCoy CW Pathogenicity enhancement of *Metarhizium anisopliae *and *Beauveria bassiana *to first instars of *Diaprepes abbreviatus *(Coleoptera: Curculionidae) with sublethal doses of imidacloprid Environ Entomol 1997 26 1173 1182

[B27] Scholte EJJ Takken W Knols BGJ Infection of adults *Aedes aegypti *and *Ae. albopictus *mosquitoes with the entomopathogenic fungus *Metarhizium anisopliae* Acta Trop 2007 102 151 158 10.1016/j.actatropica.2007.04.011 17544354

[B28] Boucias DG Stokes C Storey G Pendland JC The effects of imidacloprid on the termite *Reticulitermes flavipes *and its interaction with the mycopathogen *Beauveria bassiana* Pflanzenschutz-Nachrichten Bayer 1996 49 103 144

[B29] Quintela ED McCoy CW Conidial Attachment of *Metarhizium anisopliae *and *Beauveria bassiana *to the Larval Cuticle of *Diaprepes abbreviatus *(Coleoptera:Curculionidae) treated with imidacloprid J Invertebr Pathol 1998 72 220 230 10.1006/jipa.1998.4791 9784344

[B30] Howard AFV N'Guessan R Koenraadt CJM Asidi A Farenhorst M Akogbéto M Thomas MB Knols BGJ Takken W The entomopathogenic fungus *Beauveria bassiana *reduces instantaneous blood feeding in wild multi-insecticide-resistant *Culex quinquefasciatus *mosquitoes in Benin, West Africa Parasit Vect 2010 3 87 10.1186/1756-3305-3-87 PMC2946288 20843321

[B31] Pridgeon JW Pereira RM Becnel JJ Allan SA Clark GG Linthicum KJ Susceptibility of *Aedes aegypti*, *Culex quinquefasciatus *Say, and *Anopheles quadrimaculatus *Say to 19 Pesticides with Different Modes of Action J Med Entomol 2008 45 82 87 10.1603/0022-2585(2008)45[82:SOAACQ]2.0.CO;2 18283946

[B32] Paul A Harrington LC Scott JG Evaluation of novel insecticides for control of dengue vector *Aedes aegypti *(Diptera: Culicidae) J Med Entomol 2006 43 55 10.1603/0022-2585(2006)043[0055:EONIFC]2.0.CO;2 16506447

[B33] Sim S Dimopoulos G Dengue Virus Inhibits Immune Responses in *Aedes aegypti *Cells PLoS ONE 2010 5 e10678 10.1371/journal.pone.0010678 20502529 PMC2872661

[B34] WHO Pesticides Evaluation Scheme (WHOPES) http://www.who.int/whopes/guidelines/en/

[B35] Elandt-Johnson R Johnson NL Survival models and data analysis 1980 John Wiley and Sons, New York

