# Supplementary material for: The combination of the entomopathogenic fungus Metarhizium anisopliae with the insecticide Imidacloprid increases virulence against the dengue vector Aedes aegypti (Diptera: Culicidae)
Source: Parasit Vectors. 2011 Jan 25;4:8. doi: 10.1186/1756-3305-4-8 (PMC3037915; doi:10.1186/1756-3305-4-8)
Supplement: Additional file 1 — Figure S1 Insecticide and fungal exposure system Photograph showing the type of plastic pot used in insecticide testing and fungal infection. The filter paper shown here was impregnated by submersion in a conidial suspension for display purposes only. Mosquitoes released into the pot (plastic lid removed for clarity) had free access to resting sites not treated with fungi or insecticide. [file 1756-3305-4-8-S1.DOC]

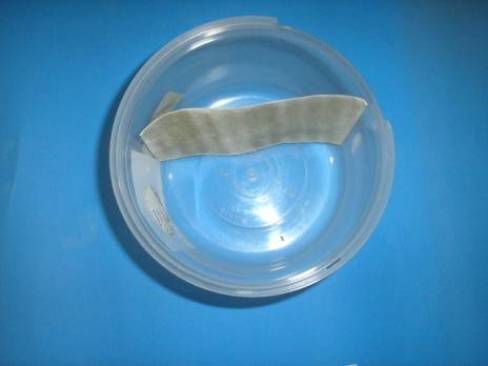


Figure S1

Title: Insecticide and fungal exposure system

Description: Photograph showing the type of plastic pot used in insecticide testing and fungal infection. The filter paper shown here was impregnated by submersion in a conidial suspension for display purposes only. Mosquitoes released into the pot (plastic lid removed for clarity) had free access to resting sites not treated with fungi or insecticide.
